# Early results of the implementation of laparoscopic major liver resection program

**DOI:** 10.1186/s12957-022-02505-5

**Published:** 2022-03-03

**Authors:** Marcin Morawski, Michał Grąt, Maciej Krasnodębski, Konrad Kobryń, Wacław Hołówko, Paweł Rykowski, Marta Dec, Małgorzata Nowosad, Wojciech Figiel, Waldemar Patkowski, Krzysztof Zieniewicz

**Affiliations:** 1 Department of General, Transplant, and Liver Surgery, Medical University of Warsaw, Banacha 1a Street, 02-097, Warsaw, Poland; 2 2nd Department of Anaesthesiology and Intensive Care, Medical University of Warsaw, 02-07, Warsaw, Poland

**Keywords:** Liver resection, Laparoscopy, Complications

## Abstract

**Background:**

Laparoscopic liver resections offer potential benefits but may require advanced laparoscopic skills and are volume dependent.

**Methods:**

This retrospective study included 12 patients who underwent major laparoscopic resection and 24 patients after open major liver resection for liver malignancy in the time period between September 2020 and May 2021. The primary outcomes were complications according to Clavien-Dindo classification and duration of hospital stay.

**Results:**

Median duration of hospital stay in laparoscopic resection group (6 days) was significantly shorter than in open resection group (8 days) (*p* = 0.046). Complications classified as grade II or higher were significantly less frequent in the laparoscopic resection group (2 patients) versus open resection group (13 patients) (*p* = 0.031).

**Conclusions:**

Although laparoscopic major liver resections should be limited to expert hepatobiliary centers and are characterized by long learning curve, this approach may offer favorable short-term outcomes even during launching a new program.

## Background

Benefits from laparoscopic approach have been shown in numerous indications [1–6]. In the recent years, rapid increase in laparoscopic liver resection could be observed owing to favorable short-term and oncological outcomes [6]. Although this minimally invasive approach entails lower surgical trauma and faster recovery, it indisputably requires expertise in hepatobiliary surgery and high technical laparoscopic skills. The length of the learning curve in major laparoscopic surgery may limit its introduction due to apprehension about intraoperative and postoperative complications and complexity of the procedure [7].

In the presented paper, we summarize early surgical outcomes of first cases of major laparoscopic liver resections performed in our department with regard to major open liver resections from the same time period.

## Materials and methods

**Fig. 1:**
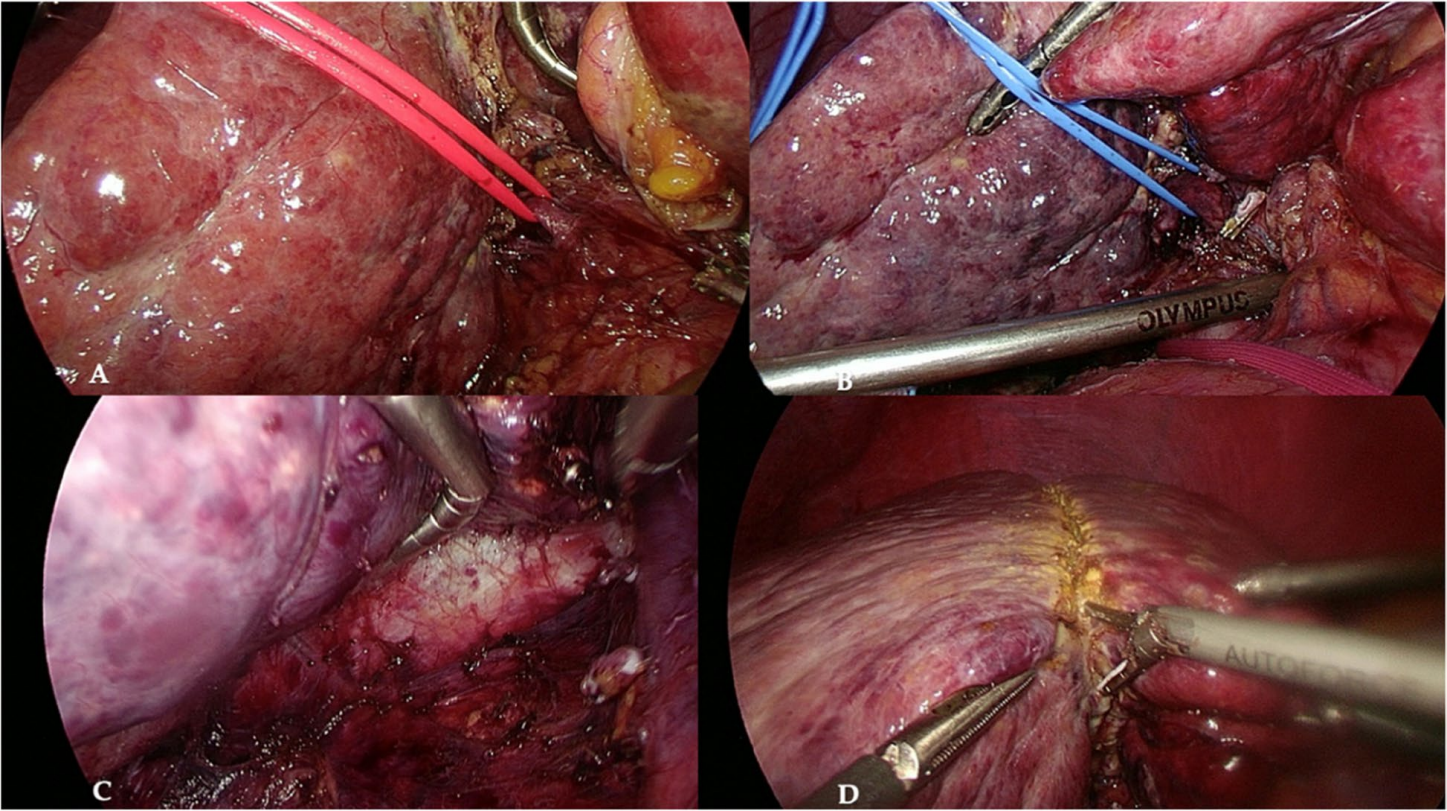
Liver resection in the cirrhotic patient with combined hepatocellular-cholangiocarcinoma. Identification of **A** right hepatic artery and **B** right portal vein branch. **C** Mobilized right liver lobe and **D** transection line

**Fig. 2:**
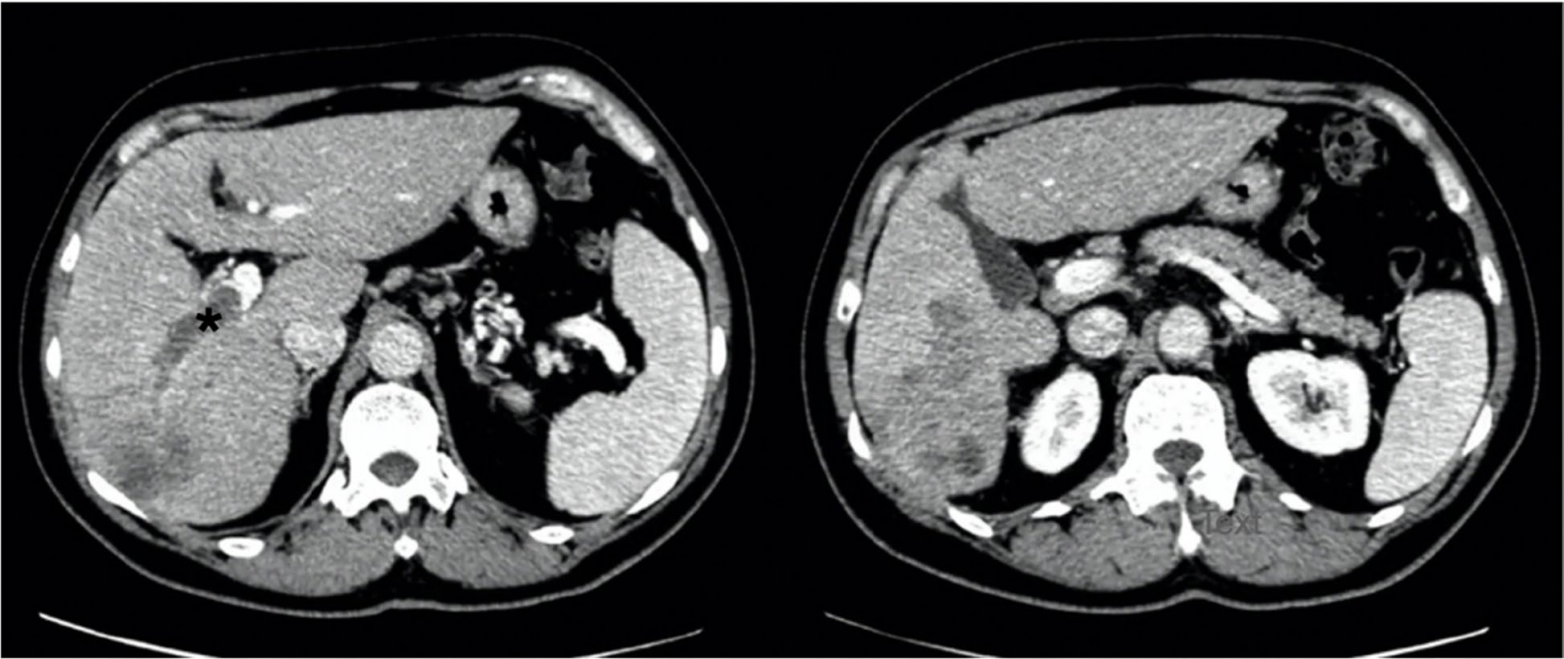
Computed tomography of the cirrhotic patient with combined hepatocellular-cholangiocarcinoma. (*) Tumor thrombus in the right portal vein branch

**Fig. 3:**
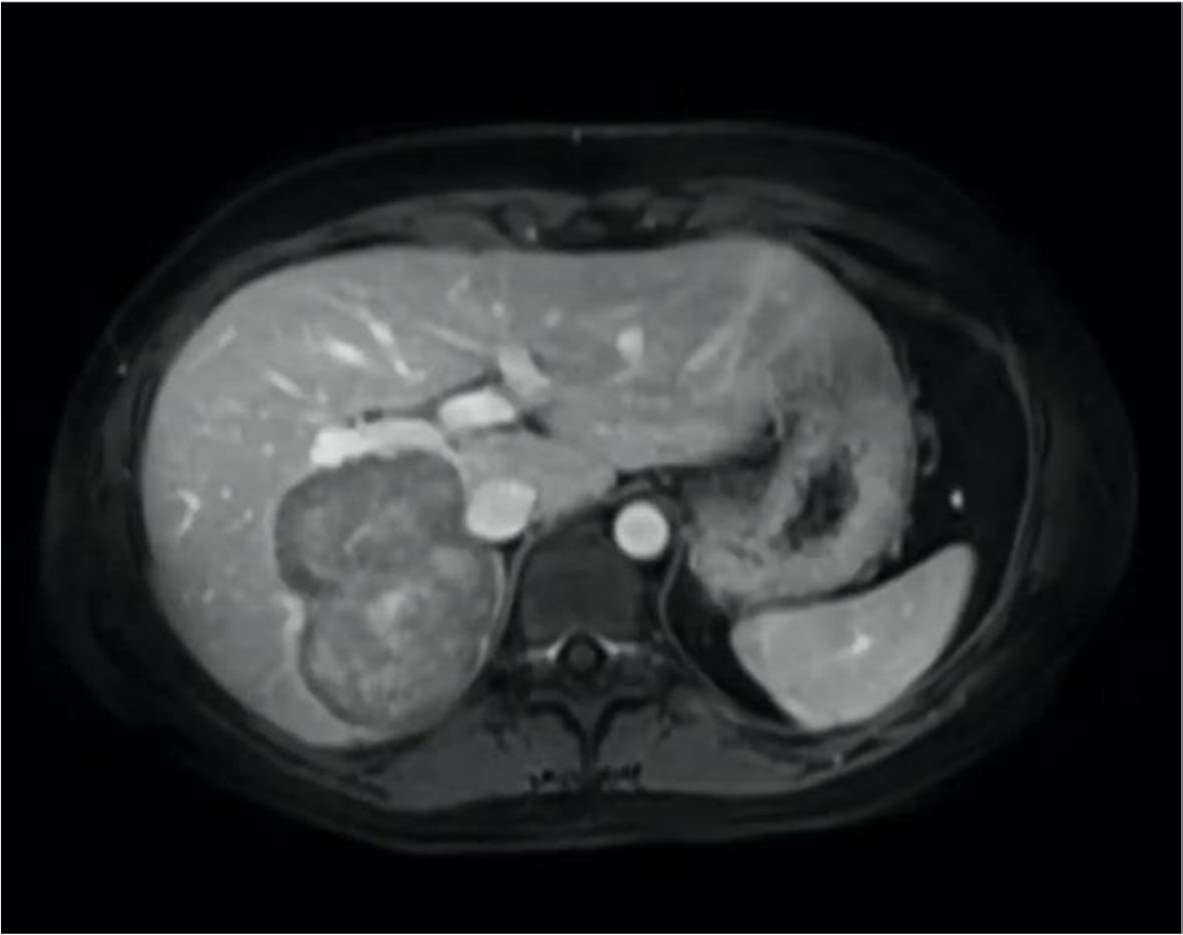
Magnetic resonance imaging of the patient with large adenoma

This was a retrospective cohort study including patients who underwent major laparoscopic liver resection (3 or more liver segments) in the Department of General, Transplant, and Liver Surgery between September 2020 and May 2021 (Figs. 1, 2, and 3).

We identified a group of 12 patients who underwent major laparoscopic liver resection in the period of interest. For each case of major laparoscopic liver resection, two cases of major open resections operated on the closest dates were found (24 patients). There were no other major laparoscopic resections that were excluded from the study.

Laparoscopic major liver resections were performed by one operating surgeon (M.G.). In brief, a total of 6 to 7 trockars were placed. First trockar was always inserted through mini-laparotomy in the midline. Abdominal cavity was insufflated to reach a pressure of 12–14 mmHg. For right hemihepatectomies, the right liver lobe was fully mobilized prior to parenchymal transection. Hemostatic clips were placed to secure right or left hepatic artery and right or left portal vein branch. Right or left hepatic duct was closed with a vascular stapler. Vascular staplers were also used for closure of hepatic veins. Parenchymal transection was carried out with ultrasonic aspirator and harmonic device. Metal clips were placed to secure larger vessels and biliary ducts on the transection surface. Extracorporeal Pringle maneuver was routinely used for parenchymal transection, except for 1 patient undergoing right hemihepatectomy in the cirrhotic liver.

Data on preoperative laboratory results, complications, duration of hospital stay, and 30-day mortality were collected. Complications were classified according to Clavien-Dindo classification [8]. Quantitative and categorical data were presented as median and interquartile range (IQR), number of cases, and percentages, respectively. The patient characteristics were analyzed and compared between groups using the Mann-Whitey *U* test and *χ*^2^ test as appropriate. The level of significance was set at 0.05. STATISTICA version 13.3, TIBCO Software Inc. (PaloAlto, CA, USA) software was used for statistical analyses.

## Results

Baseline characteristics of the study group are summarized in Table 1. In the laparoscopic resection group, there were 6 right (50%), 4 right extended (33.3%), and 2 left (16.7%) hemihepatectomies, whereas the open resection group comprised 16 right (66.7%), 3 right extended (12.5%), 3 left (12.5%), and 2 left extended hemihepatectomies (8.3%). One patient undergoing right extended hemihepatectomy in the laparoscopic group had conversion to open procedure after completion of parenchymal transection due to massive bleeding from right hepatic vein due to stapler injury. This patient, in the intention to treat principle, was retained in the laparoscopic group. Comparison of baseline characteristics between laparoscopic and open resection groups is shown in Table 1. There were no significant differences in baseline and laboratory results. The main indication for liver surgery in both groups was metastatic colorectal disease. Among other indications, there were combined hepatocellular-cholangiocarcinoma, adenoma, neuroendocrine, breast, and clear cell renal cell carcinoma metastases.

**Table 1:** Baseline characteristics of the study group and comparison of laparoscopic and open liver resection groups

Variables	Number (%) or median (IQR)	Laparoscopic liver resection	Open liver resection	*p*
Sex				0.173
Male	14 (38.9%)	7 (58.3%)	7 (29.2%)	
Female	22 (61.1%)	5 (41.7%)	17 (70.8%)	
**Age (years)**	65 (53–69)	61 (45–68)	65 (54–67)	0.208
**BMI > 25 (number of patients)**	18 (50.0%)	5 (41.7%)	13 (54.2%)	0.725
**Liver resection**				
Right hemihepatectomy	22 (61.1%)	6 (50%)	16 (66.7%)	
Right extended hemihepatectomy	7 (19.4%)	4 (33.3%)	3 (12.5%)	
Left hemihepatectomy	5 (13.9%)	2 (16.7%)	3 (12.5%)	
Left extended hemihepatectomy	2 (5.6%)		2 (8.3%)	
**Indication for liver resection**				
Colorectal metastases	20 (55.6%)	7 (58.3%)	13 (54.2%)	
Hepatocellular carcinoma	3 (8.3%)		3 (12.5%)	
Cholangiocarcinoma	6 (16.7%)	1 (8.3%)	5 (20.8%)	
Other	7 (19.4%)	4 (33.3%)	3 (12.5%)	
**Prior upper abdominal surgery**	7 (19.4%)	2 (16.7%)	5 (20.3%)	1.000
**Concomitant procedures**				
Cholecystectomy	26 (72.2%)	10 (83.3%)	16 (66.7%)	0.510
Lymphadenectomy			4 (16.7%)	
Diaphragm resection			4 (16.7%)	
Extrahepatic bile ducts resection with Roux-en-Y anastomosis			2 (8.3%)	
**Number of tumors**	1 (1–2)	1 (1–3)	1 (1–2)	0.772
**Maximal tumor diameter (millimeters)**	45 (30–75)	41 (25–61)	45 (33–80)	0.548
**Liver cirrhosis (number of patients)**	1 (2.78%)	1 (8.33%)	0 (0%)	0.333
**Preoperative laboratory results**				
White Blood Cell (10^3^/L)	6.43 (5.07–7.67)	6.43 (4.72–8.03)	6.45 (5.24–7.56)	0.933
Hemoglobin (g/dL)	13.1 (12.0–14.1)	13.0 (12.0–15.1)	13.1 (12.1–13.7)	0.523
Platelets (10^3^/μL)	213 (189–276)	208 (194–268)	222 (185–276)	0.788
Bilirubin (mg/dL)	0.44 (0.33–0.77)	0.51 (0.31–0.86)	0.44 (0.35–0.73)	0.933
Albumin (g/dL)	4.4 (4.0–4.7)	4.3 (4.1–4.7)	4.5 (4.0–4.7)	0.853
INR	1.03 (0.97–1.07)	1.04 (0.98–1.09)	1.01 (0.96–1.07)	0.449
Creatinine (mg/dL)	0.76 (0.68–0.88)	0.76 (0.64–0.87)	0.80 (0.70–0.91)	0.603
Aspartate aminotransferase (IU/L)	31 (24–41)	25 (22–34)	36 (27–44)	0.090
Alanine aminotransferase (IU/L)	27 (20–47)	23 (19–37)	29 (21–49)	0.364
**Duration of surgery (minutes)**	270 (240–540)	600 (540–690)	240 (210–285)	< 0.05*

Median duration of hospital stay in laparoscopic resection group was significantly shorter than in the open resection group (*p* = 0.046). One patient after open resection (4.2%) died within the 30-day postoperative period, whereas mortality was nil in the laparoscopic resection group (*p* = 0.473). Notably, there was a significant difference in the number of complications beyond grade I of Clavien-Dindo classification. Two patients (16.7%) in the laparoscopic group compared to 13 patients (54.2%) in the open resection group developed complications classified as grade II or more (*p* = 0.031). The groups also differed significantly with respect to the Comprehensive Complication Index (*p* = 0.039) [9]. In addition, the median duration of surgery was significantly longer in the laparoscopic group (600 min) compared to the open resection group (240 min) (*p* < 0.05).

In the laparoscopic resection group, we observed one complication of grade II and one of grade IIIb. The former patient after right hemihepatectomy required blood transfusion in the postoperative period due to anemia of 7.7 g/dL secondary to rectus sheath hematoma on postoperative day (POD) 2. The hematoma remained stable on imaging studies and did not require any additional interventions. The latter patient who also underwent right hemihepatectomy required endoscopic retrograde cholangiopancreatography (ERCP) due to biliary fistula on POD 10 and percutaneous biloma drainage on POD 17 complicated by pleural effusion requiring pleurocenteses on POD 20 and 21. Following paracenteses, the patient underwent video-assisted thoracoscopy (VATS) decortication due to stage II empyema on POD 28.

In the open resection group, 13 out of 24 patients had complications of grade II or more according to Clavien-Dindo. Data on postoperative complications are summarized in Table 2.

**Table 2:** Summary of details on postoperative hospital stay and complications according to Clavien-Dindo

	Number (%) or median (IQR)	Laparoscopic liver resection	Open liver resection	*p*
Postoperative hospital stay (days)	7 (6–12)	6 (6–8)	8 (7–14)	0.046*
**Day 4 or 5 postoperative laboratory results**				
White blood cell (10^3^/L)	7.03 (5.19–8.78)	7.18 (5.34–8.76)	6.37 (5.00–9.09)	0.934
Hemoglobin (g/dL)	10.1 (9.4–11.0)	10.0 (9.5–12.2)	10.2 (9.3–10.6)	0.804
Bilirubin (mg/dL)	0.89 (0.63–1.44)	1.06 (0.85–1.44)	0.80 (0.52–1.41)	0.120
INR	1.16 (1.04–1.30)	1.07 (1.02–1.22)	1.21 (1.06–1.33)	0.146
Creatinine (mg/dL)	0.75 (0.63–1.10)	0.76 (0.66–0.88)	0.75 (0.61–1.17)	0.908
Aspartate aminotransferase (IU/L)	114 (85–206)	179 (121–238)	101 (72–148)	0.029*
Alanine aminotransferase (IU/L)	220 (157–431)	346 (192–446)	189 (132–348)	0.090
**Intraoperative blood loss** (ml)		350 (300–400)		
**Intraoperative blood transfusions (number of patients)**	7 (19.4%)	1 (8.3%)	6 (25%)	0.234
R1 resection*	3 (8.3%)	2 (16.7%)	1 (4.2%)	0.253
**Clavien-Dindo complication grade** (number of patients)				
Grade I				
Prolonged abdominal drainage		3	5	
Surgical site infection			4	
Grade II				
Blood transfusion		1	3	
Intravenous antibiotics			6	
Total parenteral nutrition		1	2	
Grade IIIa				
Pleurocentesis			1	
Grade IIIb				
Biliary fistula		1	1	
Surgical site infection			1	
Hemorrhage			1	
Intestinal perforation			2	
Grade IV				
Portal vein thrombosis			1	
Acute kidney injury (dialysis)			1	
Grade V			1	
**Comprehensive Complication Index**		0 (0–8.7)	20.9 (0–36.0)	0.039*

*Only patients with malignant liver tumors

## Discussion

Laparoscopic liver resections are limited to few high-volume hepatobiliary centers, and due to their technical complexity and long learning curve, it may be difficult to launch such a program. Herein, based on early results of first major liver resections performed in our department, we show that introduction of minimally invasive approach in liver surgery may be measurably beneficial regarding early surgical outcomes. Given that learning curve in laparoscopic resection was estimated at 60 cases, we show that first favorable outcomes may be observed at the stage of 12 major liver resection cases [10]. However, this finding needs to be supported by further analyses on larger groups of patients less prone to the selection bias.

It has been shown that postoperative morbidity, mortality, and hospital stay are valuable outcome measures of center performance with regard to laparoscopic surgery and may provide better insight than intraoperative events (e.g., blood loss or conversion rate) [11, 12]. In our group, there was only one case of conversion due to major bleeding; however, the postoperative course in the patient was uneventful. Our results show significant reduction in median duration of hospital stay in patients undergoing laparoscopic resections. Thus, the observed significant difference in duration of surgery that can be attributed to longer learning curve may be outweighed by the shorter inpatient stay.

Our results remain consistent with conclusions existing in the literature. Kasai et al. in the meta-analysis of 917 patients report significant reduction in postoperative hospital stay between open and laparoscopic resection group and lower overall postoperative morbidity rate in favor of laparoscopic approach [13]. However, the cutoff for minor and major complications according to Clavien-Dindo was defined as grade III or more, and the authors found no significant difference between groups with respect to both levels. In terms of intraoperative blood transfusions and postoperative mortality there were no significant differences. Similarly, according to consensus conference held in Morioka laparoscopic major hepatectomy may be considered favorable with regard to length of hospital stay [14]. In addition, the experts concluded noninferiority of laparoscopy when it comes to postoperative morbidity and mortality.

Clavien-Dindo classification allows reproducible measurement of surgical complication including severity and necessity for treatment modification [15]. Although the authors of the grading system do not encourage differentiation between minor and major complications, it should be noted that all but grade I require change to standard postoperative care, including invasive approaches, and thus may be considered more severe [16]. In our group of laparoscopic liver resections, there was only one case of grade II and one case of grade IIIb complication. The patients required blood transfusion due to postoperative anemia and pleurocentesis followed by VATS due to pleural empyema secondary to percutaneous drainage of biloma, respectively.

The presented study, although limited by its retrospective nature and low number of analyzed cases, shows promising benefits for patients undergoing laparoscopic liver resections expressed by shorter hospital stay and lower prevalence of postoperative complications [17, 18]. Although the patients in case of good postoperative performance were discharged home 1 day after last drain removal, lack of unanimous discharge criteria provides a limitation to the study. Due to short observation period, we could not assess oncological outcomes in our study group. The only short-term oncological parameter—margin status—was included in the analysis and there was no significant difference in R1 resections between both groups.

In conclusion, although undoubtedly major laparoscopic liver should be limited to high-volume hepatobiliary centers and require stepwise training, it may offer early favorable outcomes on the slope of the learning curve.

## Data Availability

The datasets generated or analyzed during the current study are available from the corresponding author on reasonable request.
